# Responses to Wildfire and Prescribed Fire Smoke: A Survey of a Medically Vulnerable Adult Population in the Wildland-Urban Interface, Mariposa County, California

**DOI:** 10.3390/ijerph20021210

**Published:** 2023-01-10

**Authors:** Sumi Hoshiko, Joseph R. Buckman, Caitlin G. Jones, Kirstin R. Yeomans, Austin Mello, Ruwan Thilakaratne, Eric Sergienko, Kristina Allen, Lisa Bello, Ana G. Rappold

**Affiliations:** 1 Environmental Health Investigations Branch, Center for Healthy Communities, California Department of Public Health (CDPH), Richmond, CA 94804, USA; 2 California Epidemiologic Investigation Service Fellowship Program (Cal-EIS), Chronic Disease Control Branch, Center for Chronic Disease Prevention and Health Promotion, California Department of Public Health, Sacramento, CA 95834, USA; 3 Mariposa County Health and Human Services Agency, Mariposa, CA 95338, USA; 4 Center for Public Health and Environmental Assessment, United States Environmental Protection Agency, Research Triangle Park, NC 27711, USA

**Keywords:** wildfire, prescribed fire, smoke, adaptive capacity, vulnerable populations, prescribed burns, wildland fire, managed fire, wildland-urban-interface

## Abstract

California plans to substantially increase the use of prescribed fire to reduce risk of catastrophic wildfires. Although for a beneficial purpose, prescribed fire smoke may still pose a health concern, especially among sensitive populations. We sought to understand community health experience, adaptive capacity, and attitudes regarding wildland and prescribed fire smoke to inform public health guidance. We conducted a cross-sectional survey of medically vulnerable persons in a rural, high fire risk county (N = 106, 76% > 65 years) regarding wildfire and prescribed smoke health effects; health protective actions; information needs; and support for fire management policies. Qualitative comments were reviewed for context and emerging themes. More than half (58%) of participants reported health impacts from wildfire smoke; 26% experienced impacts from prescribed fire smoke. Participants expressed strong support for prescribed fire, although also concerns about safety and smoke. Respondents reported taking actions to reduce smoke exposure (average 5 actions taken per person), but many (47%) lacked confidence that they could successfully protect their health. Persons who were satisfied with the information received tended to be more confident in their ability to protect their health compared to those who were not satisfied (61% vs. 35%). More information was desired on many topics, including notifications about prescribed fire, health protection and exposure reduction. As California expands use of prescribed fire, the need for effective health protective communication regarding smoke is increasingly vital. We recommend seeking solutions that strengthen community resilience and address equity for vulnerable populations.

## 1. Introduction

Driven by a drier, hotter climate and historic land management practices, California has witnessed an exponential increase in wildfires, with 8 of 10 largest fires occurring since 2017 [1]. Wildfire smoke has degraded air quality for millions of persons across the state, emerging as a significant public health concern [2,3].

Recognizing this existential threat, the State of California is endeavoring to greatly increase the use of beneficial fire to as much as 400,000 acres annually by 2025 [4,5,6]. Currently, around 80,000 acres are reported as treated with prescribed fire annually [7]. Beyond the goal of preventing catastrophic wildfires, the uses of prescribed fire—intentionally ignited fires for beneficial purposes—include ecological goals such as watershed and vegetation management and wildlife habitat improvement [4]. Reintroduction of prescribed fire is aimed at restoring resilience to California’s fire-dependent natural lands following a century of fire suppression policies.

Prescribed fires are purposefully planned to limit the impact on air quality as measured by fine particulate matter (PM_2.5_) [8,9,10]. Yet, questions remain about potential health impacts, especially as effects continue to be recognized at lower exposures, particularly among sensitive subpopulations [11]. Increased migration into the wildland-urban interface (WUI) amplifies these concerns. Although previous research has found general public acceptance for prescribed burning, recent extreme wildfire seasons may have impacted public opinion, and very few studies have investigated public health impacts of prescribed fire smoke [12,13,14,15,16,17].

The goal of this study was to deepen understanding of what vulnerable communities in the WUI experience regarding wildfire and prescribed fire smoke, including health effects, residents’ capacity to protect their health, attitudes towards prescribed fire and wildfire, and factors influencing adaptive capacity and attitudes. Our survey targeted medically vulnerable adults in Mariposa County, a high fire risk area of California’s Sierra Nevada mountains.

## 2. Materials and Methods

### 2.1. Study Design and Population

Using a cross-sectional design, we surveyed participants in the program, Support and Aid for Everyone (SAFE) [18] operated by Mariposa County Health and Human Services Agency (MC), which assists persons who self-identify as having special needs in an emergency, e.g., use wheelchairs or electrical medical equipment. Mariposa County is a sparsely populated rural area with an estimated population of about 17,000 and 11.8 population per square mile which is mostly white (89%) [19]. Compared to California generally, residents are older (29% vs. 15% age 65 and older) and have lower median household incomes ($50,960 vs. $78,672) [19,20].

A questionnaire was distributed to SAFE participants on behalf of California Department of Public Health (CDPH) by MC staff allowed for anonymous participation (see Supplementary Materials). Following a modified Dillman method [21], the survey was mailed, with a phone interview option. Reminders were made by postcard and follow-up calls by MC staff. Participants could receive a $25 gift card to a local grocery or pharmacy, and all participants provided written or verbal informed consent.

We sought input from key stakeholders, including California Department of Forestry and Fire Protection (CAL FIRE), California Air Resources Board, United States Forest Service, local air districts, county health and other subject matter experts, including a prescribed burn collective.

### 2.2. Data Analysis

The questionnaire solicited demographic characteristics (age, gender, time living in county); health status; experience with wildfires and prescribed fire; and health effects of smoke. We intentionally did not ask income levels to avoid any potential deterrent effect of this more sensitive topic and also based on Mariposa County authors’ knowledge that the SAFE population is generally older and likely fixed- income. We investigated adaptive capacity by querying actions taken to reduce exposure or protect health, information needs, and current and preferred communication channels. Level of satisfaction with information received, confidence in reducing risk, and attitudes towards progressive land management policies (e.g., increasing use of prescribed fires and managed wildfires) were assessed with Likert scales. Chi-square tests were used to investigate factors associated with confidence in ability to protect health. Multivariate logistic regression using forward selection based on an a priori set of variables was used to estimate odds ratios for support and concerns regarding land management policies. We hypothesized the following factors may influenced attitudes, although we were agnostic as to the direction: having been previously affected by wildfires; having worse health status (as measured by a greater number of medical conditions); and whether someone was a long-time resident. Although we considered age, because of potential collinearity with length of residence in the county, we chose length of residence as the more useful variable, especially since most of the study population was older. Quantitative analyses were conducted in R version 4.2.1 (R Core Team 2021, Vienna, Austria). Open-ended comment boxes provided context and were evaluated for emerging themes.

## 3. Results

### 3.1. Population Characteristics and Health Status (Table 1)

The total number of persons surveyed was 106, a response rate of 40%. A family member or caregiver assisted 15% of respondents with the survey; 4% were conducted by phone. The majority of respondents were female (73.1%); most were age 65 or older (80.0%); and many were long-term residents (41.7% have lived in the county for over 20 years). Most (86.1%) participants had one or more chronic health conditions; the most commonly reported was hypertension (58.4%).

“I’m on life support machines at night when I sleep.”

### 3.2. Experience with Wildfire, Prescribed Fire and Health Impacts

Most participants were familiar with wildfire and prescribed fire, with 76% reporting being affected by wildfire. While 9% lacked familiarity with prescribed fire, others (19%) reported that they have used prescribed fire.

Over half of our study population felt their health had been affected by wildfire smoke (58%) and 26% felt their health was affected by prescribed fire smoke. Over a third of respondents (38%) reported seeking care for smoke-related health symptoms. One participant shared that anxiety about smoke led to cardiovascular symptoms.

### 3.3. Adaptive Capacity

#### 3.3.1. Confidence in Reducing Health Risks

A slight majority perceived themselves as confident in reducing health impacts of smoke (53%), leaving a meaningful proportion that did not. Some described themselves as having some knowledge of what actions to take but lacking confidence in being able to reduce smoke impacts on their health (39%). Others indicated they had little knowledge of what actions to take (12%).

“Help for elderly with breathing problems like a place to go until prescribed fire is finished... Specifically elderly like me with oxygen intake (daily) also need transportation to one place to another.”

#### 3.3.2. Actions Taken (Table 2)

Overall, the majority of participants were taking action to protect their health from smoke (95%), and 56% reported taking action to protect themselves from prescribed fire smoke specifically. On average participants reported taking over five health protective actions. For wildfire smoke, the most common health protective actions were avoiding outdoor recreation, avoiding daily activity, and staying indoors. The next most common was wearing a face mask (46%); however, only 14% reported wearing an N95 or other respirator. Another widely used action was to run air conditioning. Air cleaners and do-it-yourself (DIY) methods using a box fan were less utilized risk reduction methods.

Many respondents commented on their attempts to create clean air in their homes, some trying DIY methods, but others identifying a need for commercial air cleaners. Some respondents’ experience with masks was positive, but others were unable to wear a mask. Other responses indicated residents were utilizing air quality information to guide their activities.

Similar types of protective actions were reported in response to prescribed fire smoke, but a smaller proportion of respondents reported taking these measures. Although more participants reported leaving the area for wildfire smoke (43%), some participants left the area for prescribed fire smoke (13%).

#### 3.3.3. Communications (Table 3)

Participants were interested in every type of information suggested about prescribed fire. The majority of individuals preferred one week’s advance notice. The next most desired type of information was time of day when smoke would be present (58%) and details, e.g., size, reasons, who is conducting it.

Desire for detailed prescribed fire information was followed closely by interest in health topics, such as health risks for persons with medical conditions (50%). Practical information was also sought, such as where to buy respirators, how to use them, how to make a clean room and homemade filter, where to find a clean air shelter (25–39%). One person asked to see a video showing how to attach a filter to a box fan. Information on relocation was of strong interest (39%).

Preferences for news sources shifted between past and future. Local news sources (e.g., television, newspapers) were the most frequently utilized information source (41%) in the past. Government sources were most frequently requested for future information (44%), and interest in future use was increased across all agency sources. Few participants reported using social media (2–17%), though there was increasing desire to obtain information on Twitter and online forums, as well as from prescribed burn associations in the future. Overall, 19% were dissatisfied with the information they receive about prescribed fire, either disagreeing (11%) or somewhat disagreeing (7%) with the statement that they were satisfied with the information they currently receive. Respondents showed the strongest desire for information from their local Mariposa County public health staff in the future (46%).

People reported varied sources that they received and desired information from, including apps such as NextDoor. Mariposa County Sheriff’s Office was mentioned multiple times, suggesting its role as a trusted source and the perceived value of receiving phone calls among this population. Non-internet-dependent sources were considered desirable as people mentioned the library and mailings. Even if residents had internet, they still requested texts because of internet unreliability; residents desired both more information and more reliability.

#### 3.3.4. Factors Influencing Adaptive Capacity

Among persons who were satisfied with the information received about prescribed fire, we found a higher percentage of persons who felt confident in their ability to protect their health, a marginally statistically significant difference (61% confident among those satisfied with information vs. 35% confident among those not satisfied with information; *p* = 0.06). Those who were less confident in their ability to protect health were more likely to report taking action for wildfire smoke (93% vs. 72% for persons confident, *p* = 0.01); with a similar but non-significant trend for prescribed fire smoke (*p* = 0.43). Qualitative responses revealed the crucial importance of community, family, and agencies (Table 4).

### 3.4. Attitudes toward Prescribed Fire and Managed Fire

Overall, an overwhelmingly large majority of respondents agreed with conducting prescribed fire to prevent large wildfires (86%) as well as for ecological purposes (78%). When asked specifically about the policy of increasing prescribed fire, 69% were in support and 25% stated that they would support if they could have more information on certain concerns. Only 4% of the group surveyed said that they did not support increasing prescribed fire. When asked if they support allowing natural or accidental wildfires to burn in a controlled, beneficial way, 63% agreed, 27% supported with qualifications, and 7% disagreed.

Although comments by respondents generally expressed strong positive opinions about prescribed fire, concerns were expressed in tandem (Table 4). Some supported prescribed fire but were opposed if conducted during drought conditions; another questioned why it could be allowed on windy days or flatly disagreed with any prescribed fire in Mariposa County. In comments addressing managed fire, one noted they would trust the fire department’s knowledge, and another would support if the resources were available to keep it under control.

Factors Influencing Attitudes Regarding Fire Management Policies (Table 5)

Demographic factors influenced support for fire management policies. People who lived in the county more than 20 years were less likely to support increasing prescribed fire or managed fire. Being previously affected by wildfire and having two or more medical conditions were associated with increased support for these policies, although these measures were not statistically significant. Slightly greater support for increasing prescribed fire was seen among persons who felt confident in protecting their health (94% supporting) compared to persons lacking confidence (91%). Concerns about prescribed fire getting out of control or smoke were associated with longer time living in the county, being previously affected by wildfire, and having more medical conditions, although not statistically significant.

### 3.5. Recommendations from Participants and Emerging Themes

Respondents wanted help with vegetation management, including mechanical fuel reduction methods, mentioning the Natural Resources Conservation Service, which supports landowners’ conservation efforts [24]. Other requests included weed clearing near town and more year-round firefighters.

One Health, an interdisciplinary concept of the interconnectedness of human and animal health [25] emerged as a theme as residents own livestock and described strong emotional attachments to pets; they must consider how to evacuate with them as well as protect them from smoke. Worry about harm to wildlife from prescribed fire was also raised.

## 4. Discussion

Most respondents in this medically vulnerable WUI-population indicated their health is impacted by wildfire smoke. More surprisingly, a significant proportion also identified prescribed fire smoke as affecting their health. Participants often shared feelings of anxiety related to wildfires and even prescribed fire. The health impacts described in our study extend beyond what is typically discerned from health care utilization analyses, as residents’ qualitative comments revealed a much more holistic picture of the toll taken on their lives from wildfires, prescribed fires and resulting smoke, including details of the emotional impacts, disruption to daily routines, the ways they have responded, and their reliance on social networks.

Although residents may not be accessing medical care for symptoms during smoke events, they overwhelmingly reported taking multiple actions to protect their health from smoke, with 88% reporting taking some action for wildfire smoke and over half reporting actions taken specifically for prescribed fire smoke. However, their choice of actions may reflect knowledge shortcomings or lack of access to more effective options. For example, many people reported using face masks for smoke protection (46% for wildfire and 31% for prescribed fire smoke), which do not protect against the smaller components of smoke which pose the greatest health risks [26]. Furthermore, fewer reported using more effective—though more costly—options like air purifiers. Despite their efforts, only slightly more than half of respondents overall perceived themselves as confident in being able to protect their health against smoke effects and nearly half described themselves as not confident, suggesting both a compelling need and opportunity for public health agencies to provide this education and seek more ways to provide support.

Because prescribed fires are planned, they often offer an opportunity for better preparation and mitigation of the impacts of smoke. Our findings are consistent with previous research on the effectiveness of local, trusted sources and personal interactions for communicating prescribed fire information, along with the desirability of personalized communications such as phone calls [12]. Effectiveness of this messaging will be increased if notification is paired with clear information on actions to take. Respondents rated their county health agency highest among desired information sources; this highlights the value of public health officials’ participation in message delivery. Family and friends also played an important role in providing support and health guidance; reaching them can be an efficacious strategy. In other WUI community research we conducted, participants recommended providing education via community events or festivals organized collaboratively by community and local fire departments [27].

Information sought by residents ranged from practical questions, such as how to find Air Quality Index (AQI) or smoke forecasts on their smart phone or computer, to more general information about smoke health effects and prescribed fire education. Fortunately, scientific advances are increasing accuracy in smoke modeling and forecasting, and more tools are available [28,29,30,31]. AirNow’s Fire and Smoke Map provides timely information on wildfire smoke, and many individuals are using local air quality sensors [32,33,34]. Mobile apps such as California’s Smoke Spotter can provide information on prescribed fire smoke, which can help residents plan their day as well as distinguish if smoke is from a prescribed fire rather than a dangerous wildfire [35]. Air Resource Advisors can be deployed to wildfires to support communicating smoke impacts [36].

While these are excellent tools, the challenge is to make them widely known and accessible to the public. One of the greatest hurdles is reliable internet service, especially in rural areas. Mass phone calls could circumvent this infrastructure weakness, providing time-sensitive notifications such as changes in planned prescribed fires, alerts for wildfires and smoke, even functioning during power safety shutoffs due to wildfire risk. The considerable variety in sources of information sought underscores the need for multiple channels as well as the need for non-internet dependent options, all of which could alleviate dissatisfaction with information received.

Our study was unique in its ability to access hard-to-reach individuals at high risk for smoke health impacts by use of the SAFE program, a known, medically vulnerable population. Limitations include the response rate of 40%. However, this is within the range in other prescribed fire attitude surveys (6–55%) and avoids reliance on convenience samples which are often used for public health messaging surveys [37,38,39,40,41,42,43,44]. Still, selection bias may have occurred. It is possible that those who had stronger opinions on the use of prescribed fire, whether positive or negative, responded at a higher rate. Additionally, because we sampled a medically vulnerable population, health status may have influenced who responded, although based on the Mariposa County authors’ familiarity with SAFE individuals, the profile of those who responded appeared fairly typical of the SAFE population overall. Comparison with non-respondents was not possible and we cannot ascertain how these potential limitations may have impacted our results. Nevertheless, we believe our study generated data relevant to vulnerable WUI populations, if not the general public.

We found that an exceptionally high proportion of our respondents support prescribed fire, consistent in direction but stronger than the level of support typically seen in other surveys [12]. The tendency for the same factors that were associated with increased support for prescribed fire policies, i.e., being previously affected by wildfires and having more medical conditions, to also be associated with concerns about both smoke from prescribed fire and concerns about control, underscores the importance of avoiding simplistic interpretations of public opinion. The finding that longer-term residents were less supportive of prescribed fire suggests greater apprehension among this population and calls for particular attention to communications with this group.

Insights into the exceptions to the general high regard for prescribed fire may be gleaned from some of the qualitative findings, which suggest possible misconceptions that may be undermining the level of prescribed fire acceptance. For example, “It is frightening but I think it needs to be done but not during drought conditions.” In reality, favorable fuel and weather conditions for prescribed fire may occur even within “wildfire season,” yet the public may be unaware of the carefully considered safety criteria that is applied and oppose these decisions. Lastly, although the segment of the population who are uncomfortable with prescribed fire may be a minority, this should not be considered negligible. Addressing their concerns will be invaluable, as they are typically shared by the many residents who do support prescribed fire.

Research continues to advance our understanding of the effectiveness of interventions. Smoke protection recommendations such as staying indoors and using air filtration are supported by the literature, although with limitations [43,45]. Our findings reveal economic barriers, such as the many people reporting use of DIY air filters, an option designed to help persons without enough resources to purchase a commercial air filter. While it is positive that use of such low-cost alternatives has increased, as wildfires and prescribed fires continue, additional sustainable solutions will be important.

Several jurisdictions are exploring programs to support residents with assistance in ventilation system upgrades and portable air cleaners [46]. One filter loaner program resulted in opportunities for prescribed fire education, as well as prompting residents to purchase their own filters [46]. Guidance and CME trainings are available to public health officials and clinicians, who play a key role in counseling patients in protecting their health in preparation for fire smoke [47,48]. Still, the danger of heat co-occurring with smoke will require technological solutions to resolve. Some research is underway to develop affordable and effective filtration systems that also have cooling capabilities [49]. Socioeconomic factors must be considered as they influence access to mitigation measures and thus adaptive capacity, highlighting the importance of low-cost intervention options.

## 5. Conclusions

In our study, we learned how vulnerable residents are striving to protect their health from smoke. Our findings suggest that when people feel they possess the knowledge and tools to reduce their smoke exposure, they feel more confident in their ability to protect their health. As use of prescribed fire increases, effective outreach and education on how to mitigate the impacts of smoke are essential.

Addressing the wildfire threat facing California will require an all-of-the-above approach to mitigation, including the use of prescribed fire. For this effort to be successful, collaboration between communities of interest—public health, air quality, fire protection, land management, environmental and health organizations—will be increasingly critical in protecting the public’s health from wildfire and prescribed fire smoke. Scientific and technical innovations are urgently needed, but knowledge and resources must reach those who are vulnerable. Engaging the community and building confidence will foster the health, social and emotional resilience that can ultimately enhance the quality of life for populations facing wildland smoke challenges.

## Figures and Tables

**Table 1:** Characteristics of survey participants in a medically vulnerable adult population (N = 106) ^a^ in Mariposa County, California, 2021 ^b^.

Characteristic—No. (%)	Study Population		Mariposa County ^i^ [22,23]		
Age group ^c^					
50–64	20	(20.0)		4157	(28.7)
65–79	41	(41.0)		3816	(26.4)
80+	39	(39.0)		1065	(7.4)
Gender					
Female	76	(73.1)		7052	(48.7)
Male	28	(26.9)		7432	(51.3)
Time in county					
<10 years	26	(25.2)			
10–19 years	34	(33.0)			
20+ years	43	(41.7)			
Health conditions					
Allergies ^d^	39	(38.6)			
Asthma	29	(28.7)			
COPD	29	(28.7)			
Other respiratory disease ^e^	16	(15.8)			
Hypertension ^f^	59	(58.4)			
Metabolic condition ^g^	20	(19.8)			
Other condition ^h^	32	(31.7)			
Number of conditions					
No conditions	14	(13.9)			
1 condition	25	(24.8)			
2 or more conditions	62	(61.4)			

Abbreviations: COPD, chronic obstructive pulmonary disease. ^a^ Because of missing values, the number of observations in each characteristic category may vary. ^b^ Data source: a cross-sectional mail-based survey conducted by the California Department of Public Health. ^c^ Ages < 50 not displayed due to confidentiality protections. ^d^ Allergies or other conditions to the upper respiratory tract, eyes, and ears. ^e^ Respiratory disease that is not COPD or asthma. ^f^ High blood pressure or other heart disease. ^g^ Type 2 diabetes, metabolic syndrome, or obesity. ^h^ Conditions that were not listed, or respondent preferred not to specify condition. ^i^ Percentages calculated out of population 18 years and older.

**Table 2:** Actions taken to reduce risk reported by respondents in a survey of a medically vulnerable adult population regarding wildfire and prescribed fire smoke (N = 106), Mariposa County, California, 2021 ^a^.

Risk Reduction Method Used	Wildfire Smoke		Prescribed Fire Smoke	
	No. (%)		No. (%)	
Face mask	49	(46.2)	33	(31.1)
N95 or other respirator	15	(14.2)	8	(7.5)
Air cleaner or purifier	31	(29.2)	15	(14.2)
Homemade air cleaner	12	(11.3)	4	(3.8)
Avoided daily activity	75	(70.8)	39	(36.8)
Avoid outdoor recreation	80	(75.5)	42	(39.6)
Stayed indoors	79	(74.5)	39	(36.8)
Left affected area	45	(42.5)	14	(13.2)
Ran air conditioning	43	(40.6)	25	(23.6)
Used additional medication (e.g., inhaler)	28	(26.4)	18	(17.0)

^a^ Data source: a cross-sectional mail-based survey conducted by the California Department of Public Health.

**Table 3:** Communication and information needs and preferences regarding prescribed fire in a survey of a medically vulnerable adult population (N = 106) in Mariposa County, California, 2021 ^a^.

Advance Notice Needs—No. (%)										
1 week	77		(72.6)							
1–2 days	55		(51.9)							
**Information preferred on prescribed burns—No. (%)**										
Time of day		61	(57.5)	How to make a clean air room					29	(27.4)
How to find AQI information		25	(23.6)	How to make an at-home air filter					26	(24.5)
Rx detailed information		59	(55.7)	Where to find a clean air shelter					33	(31.1)
Health information		53	(50.0)	Relocation information					41	(38.7)
Where to buy respirators		41	(38.7)	Other protective actions					28	(26.4)
How to use respirators		28	(26.4)	General information on Rx burns					34	(32.1)
**Information sources ^b^**				**Past ^c^—No. (%)**			**Future ^d^—No. (%)**			
Local news										
Local television				43		(40.6)	34	(32.1)		
Local radio				12		(11.3)	17	(16.0)		
Local newspaper				36		(34.0)	29	(27.4)		
Local website				24		(22.6)	24	(22.6)		
Government										
Local air pollution control district				10		(9.4)	17	(16.0)		
Mariposa County Health Department				27		(25.5)	49	(46.2)		
California Air Resources Board				6		(5.7)	13	(12.3)		
US Forest Service				17		(16.0)	26	(24.5)		
US Bureau of Land Management				5		(4.7)	17	(16.0)		
CAL FIRE				24		(22.6)	36	(34.0)		
AirNow.gov				1		(0.9)	8	(7.5)		
Social media										
Facebook				18		(17.0)	16	(15.1)		
Twitter				2		(1.9)	6	(5.7)		
Online forum				5		(4.7)	12	(11.3)		
Other										
Private landowner				10		(9.4)	16	(15.1)		
Community prescribed burn associations				4		(3.8)	18	(17.0)		
Nixle				15		(14.2)	16	(15.1)		
Road sign				21		(19.8)	20	(18.9)		
**Information source used and desired**				**Past [N = 280]—No. (%)**			**Future [N = 374]—No. (%)**			
Local news				115		(41.1)	104	(27.8)		
Government				90		(32.1)	166	(44.4)		
Social media				25		(8.9)	34	(9.1)		
Other				50		(17.9)	70	(18.7)		

Abbreviations: Rx, prescribed fire; AQI, Air Quality Index; CAL FIRE, California Department of Forestry and Fire Protection. ^a^ Data source: a cross-sectional mail-based survey conducted by the California Department of Public Health. ^b^ Total percent not equal to 100% due to participants being able to select multiple responses. ^c^ Information sources used in the past for prescribed fire notifications. ^d^ Information sources preferred in the future for prescribed fire notifications.

**Table 4:** Qualitative data on residents’ experiences with wildfires and prescribed fires from a survey of a medically vulnerable adult population, Mariposa County, California, 2021 ^a^.

Topics	Illustrative Quotes
Impact of wildfire and prescribed fire	
Respiratory symptoms	“All smoke, especially that containing ash, affects my breathing”
Anxiety and cardiovascular	Respondent indicated that anxiety from smoke led to cardiovascular symptoms.
Controlling symptoms	“I used what I need [for] light smoke...Heavy smoke, I will try to find a place to go. I let my breathing determine/tell me what I need. So far so good.”
Smoke from wildfire	“Several years ago, my house filled with smoke every day for 3+ weeks”
Evacuation	Respondent evacuated 5 times from different places
No impact from prescribed fire	“No prescribed fire in Mariposa. Some in Yosemite National Park which did not affect me.”
Adaptive actions—limitations and challenges	
Information access	“Mrs. X has no radio, computers, just TV. She depends on a nearby family member as a caregiver to keep her informed.
Shelter for prescribed fire	“Help for elderly with breathing problems like a place to go until prescribed fire is finished…Specifically, elderly like me with oxygen intake (daily) also need transportation.”
Physical limitations	“Unable to wear a mask”“Being handicapped my mom has to take me to a friend’s house but only when evacuated for wildfire.
Knowledge	One person indicated they had little knowledge of what actions to take to protect their health from smoke, but added, “but I live with my family and they do.”
Air filters	“Just purchased 2 Dyson air filters. Last year the smoke was in my house, the homemade air filter was not sufficient.”“DIY air filter has worked in the past.”
Heat and smoke	“I only have a swamp cooler for cooling, which brings smoke into my house.”
Fireproofing	“I feel terrified each summer because of the threat—my perimeter …is hard to maintain—such tall weeds”
Communications about wildfire and prescribed fire	
Advance notice	“I am used to neighbors burning but large-scale events are more frightening if not forewarned.”
Friends and family	“Friends text me.”
Mailings	“Mailings are most helpful.”
Internet	“I get most of my information from the internet and online sources.”
Attitudes toward prescribed fires	
Favor health benefit	“We know there’s a need to move ahead with this…We will be rewarded health-wise in the end.”
Support forest management	“I strongly believe we need to thin out and manage our forest understory more aggressively. WE HAVE NOT TAKEN THIS RESPONSIBILITY SERIOUSLY! This includes logging where necessary.”
Concern—drought conditions	“It is frightening but I do think it needs to be done but not during drought conditions.”
Concern—windy conditions	“I worry about control with these fires. Why sometimes it is done on a windy day and it gets out of control.”
Concern—timing and wildfires	“I would prefer them to be in off season, don’t want them done during a wildfire in adjoining area further affecting air quality.”
Opposition	“You would be out of your mind to do a prescribed fire in Mariposa.”

^a^ Data source: a cross-sectional mail-based survey conducted by the California Department of Public Health.

**Table 5:** Estimated odds ratios ^a^ for support and concerns regarding land management policies of increasing prescribed burns and managed wildfires, in a survey of a medically vulnerable adult population in Mariposa County, California, 2021 ^b^.

Support for Land Management Policies				
	Prescribed Burn Increase ^c^		Managed Wildfires ^d^	
	OR	95% C.I.	OR	95% C.I.
Time living in county (>20 years) ^g^	0.12	0.01, 0.82	0.16	0.02, 0.72
Previously affected by wildfire ^h^	3.49	0.55, 22.20	2.90	0.61,13.40
2 or more medical conditions ^i^	1.23	0.19, 7.61	1.06	0.22, 4.72
**Concerns for Prescribed Burning**				
	**Concerns of Prescribed Fire Control ^e^**		**Concerns of Prescribed Fire Smoke ^f^**	
Time living in county (>20 years)	2.29	0.88, 6.44	1.80	0.72, 4.65
Previously affected by wildfire	2.79	0.98, 8.10	2.41	0.86, 6.96
2 or more medical conditions	1.82	0.72, 4.61	2.01	0.81, 5.05

Abbreviations: OR; odds ratio, 95% C.I.; 95% confidence interval. ^a^ Forward selection was used to select the predictors included in the model. ^b^ Data source: a cross-sectional mail-based survey conducted by the California Department of Public Health. ^c^ Support for prescribed increase policy in California was defined as fully supporting or support with reservations. ^d^ Support for managed wildfires in California was defined as fully supporting or support with reservations. ^e^ Concerns about prescribed fire control were assessed on a Likert scale and then recategorized into those who agreed and somewhat agreed (concerned) and those who disagreed, somewhat disagreed, or were neutral (others). ^f^ Concerns about prescribed fire smoke were assessed on a Likert scale and then recategorized into those who agreed and somewhat agreed (concerned) and those who disagreed, somewhat disagreed, or were neutral (others). ^g^ Referent group is those who reported living in the county for less than 20 years. ^h^ Referent group is those who did not report being previously affected by wildfire.*^i^* Referent group is those who reported fewer than two medical conditions.

## Data Availability

Raw data from this study will not be made publicly available to protect confidentiality. Data analysis files are available from the corresponding author upon reasonable request.

## Supplementary Materials

The following supporting information can be downloaded at: https://www.mdpi.com/article/10.3390/ijerph20021210/s1, Public Health Impact of Prescribed Fire (PHIRE) Survey.
