# Supplementary material for: Responses to Wildfire and Prescribed Fire Smoke: A Survey of a Medically Vulnerable Adult Population in the Wildland-Urban Interface, Mariposa County, California
Source: Int J Environ Res Public Health. 2023 Jan 10;20(2):1210. doi: 10.3390/ijerph20021210 (PMC9858942; doi:10.3390/ijerph20021210)
Supplement: Supplementary file 1 [file ijerph-20-01210-s001.zip › ijerph-2065395-supplementary.pdf]

PUBLIC HEALTH IMPACT OF PRESCRIBED FIRE (PHIRE) SURVEY

#___________

Please review the Consent Form on the next page, and if you agree to take the survey, mark your answers and return the consent form and survey in the enclosed envelope. Thank you for your interest!

California Department of Public Health

Consent to Participate in Research about Prescribed Fires – Mariposa County Survey of S.A.F.E. participants

Title of study: Public Health Impacts of Prescribed Fire ("PHIRE Study")
Investigator: Sumi Hoshiko, MPH, California Department of Public Health

Introduction and Purpose: You are being invited to join a research study by filling out a survey. The purpose of the study is to understand community health impacts and concerns about prescribed fires and wildfires. Participation is completely voluntary and anonymous. All participants will receive a $25 cash gift card.

Procedure: If you agree to participate in this research, you will be asked to fill out a survey. The topics will include attitudes and experiences about prescribed fire and wildfires, how you would like to receive notifications about planned prescribed fires, what actions you might take to protect your health from smoke, and needs related to prescribed fire events and demographic information. The survey would take about 10-15 minutes.

Benefits: There is no direct benefit to you from taking part in this study. The investigators hope that the research will increase knowledge about how people can reduce exposures and protect their health, and as well as improve communications around prescribed fires and wildfires, protecting health and saving lives across California communities.

Risks: Some of the questions might make you feel uncomfortable. You do not have to answer any of the questions you do not want to answer. The researchers have extensive procedures to protect the security of all information collected.

Confidentiality: The survey is anonymous and you will be tracked by a number only, not your name. All information collected will be kept confidential. Results of the research may be presented at scientific meetings or published in journals. Most questions are multiple choice, but comments written in the survey may be cited although without any names. The risk of mistakenly releasing information about your anonymous answers or your participation in the survey is small, but the researchers cannot guarantee that this will never happen. Your answers will not be shared with other researchers.

Compensation: To thank you for participating in this study, you will receive a $25 gift card to Pioneer Market or RiteAid (your choice). Please allow a month for your gift card to arrive.

Your Participation is Completely Voluntary. The survey is not part of the S.A.F.E. program. Whether you fill out the survey or not, you will not lose any rights or benefits you would otherwise be entitled to in S.A.F.E. or any other program. You may stop participating at any time. You don't have to answer any questions you prefer not to. You don't have to sign any document you don't want to.

Questions: If you have any questions please feel free to contact the Principal Investigator, Sumi Hoshiko, MPH, at the California Department of Public Health: (510) 620-3620 or Sumi.hoshiko@cdph.ca.gov. You may also ask the California Health and Human Services Agency, Committee for the Protection of Human Subjects, about your rights as a research participant: (916) 326-3661.

If you agree to participate in this research, please check the box and return this form with your survey in the envelope provided. A separate copy of this form is provided for your personal records.

_____ ***I agree to participate in this research and have received a copy of the Participant's Bill of Rights for Non-Medical Research. (If you do not agree to participate in the research, no further action is needed.)***

Date___________________

NOTE: If you would prefer to have someone ask you the questions over the phone, please call us at (510) 890-2567 and ask about the PHIRE Study (pronounced "Fire"). If it is difficult for you to fill out the survey, you may have someone help you, such as a household member.

Begin the survey here!

Please be sure to select your gift card preference at the end of this survey.

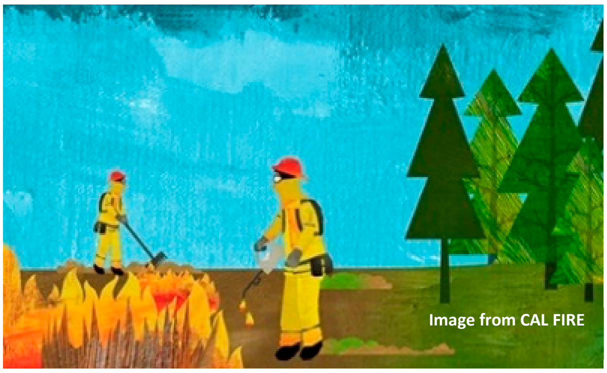

We are examining how individuals respond to smoke from wildfires and prescribed fires.

What is ***prescribed fire***?

Prescribed fires are controlled burns that are conducted for many purposes. They can reduce the risk of larger, more destructive wildfires, improve forest health and ecology, make rangeland better for grazing, or improve habitats. Prescribed fires vary in size and type. Some are applied to many acres of land, others to burning piles of cut vegetation.

Prescribed fires may be called prescribed burns, controlled burns, hazard reduction burns, vegetation management burns, planned burns, or rx burns. They may be conducted by agencies such as Cal FIRE or US Forest Service, private landowners, or community associations.

The answers should be about the person who received the survey (S.A.F.E. member). If you need assistance completing this survey, or if you would prefer to have someone ask you the questions, please call (510) 890-2567. If another person will be helping you with the survey, please select that option below.

☐ I am completing the survey for myself
☐ Someone is assisting to complete the survey on my behalf
☐ I called the research team at the number above for assistance completing this survey

PART I - WILDFIRE

1. Check all that describe your experience with ***wildfires*** in Mariposa County in the past 5 years.

☐ I have been directly affected by wildfires, such as evacuated.
☐ I felt my health was affected by the smoke.
☐ I took actions to reduce my exposure to smoke, such as staying indoors, wearing a mask, using a home air filter, etc.
☐ I was aware of a wildfire in my area, but I did not need to take any actions.
☐ I have no experience with wildfire where I currently live, but I experienced wildfires in another area.
☐ I have no experience with wildfires.
☐ I don't know.

Please add any comments if you need to clarify your response:

PART II – PRESCRIBED FIRE

2. Check all that describe your experience with ***prescribed fire*** in Mariposa County in the past 5 years.

☐ I am not familiar with prescribed fire.
☐ I was aware of a prescribed fire in my area but was not directly affected and did not need to take any actions.
☐ I have taken action to reduce my exposure to smoke from prescribed fire such as staying indoors, wearing a mask, and using a home air filter, etc.
☐ I felt my health was affected by smoke from prescribed fire.
☐ I have used prescribed fire on my property.
☐ Other (please note below).
☐ I don't know.

Please add any comments if you would like to clarify your response:

3. Please circle the number that shows how much you agree or disagree with each statement.

Statements	Disagree	Somewhat disagree	Neutral	Somewhat agree	Agree
I'm satisfied with the information I receive about prescribed fires in my area.	1	2	3	4	5
I'm concerned about prescribed fire because there is a danger the fire could get out of control.	1	2	3	4	5
I'm concerned about prescribed fire because of the smoke which could harm *my health*.	1	2	3	4	5
More prescribed fires should be done to improve forest health, for example by reducing invasive plants.	1	2	3	4	5
More prescribed fires should be done to reduce the risk of large wildfires.	1	2	3	4	5

4. Please indicate how you have been notified about prescribed fire, and how you would like to receive future notification. **Check all that apply**.

Notification source	I have received information from here **in the past**	I would like to receive information from here **in the future**	I am **not familiar** with this source
Local news (television)	☐	☐	☐
Local news (radio)	☐	☐	☐
Local news (newspaper)	☐	☐	☐
Local news (online news)	☐	☐	☐
Local air pollution control district	☐	☐	☐
Local Mariposa Public Health Department	☐	☐	☐
California Air Resources Board (CARB)	☐	☐	☐
US Forest Service	☐	☐	☐
Bureau of Land Management	☐	☐	☐
CAL FIRE	☐	☐	☐
Private landowner	☐	☐	☐
Community prescribed burn associations	☐	☐	☐
AirNow.gov	☐	☐	☐
Facebook	☐	☐	☐
Twitter	☐	☐	☐
Nixle	☐	☐	☐
Roadway sign	☐	☐	☐
Online forum (for example, Nextdoor or Neighbor App)	☐	☐	☐

Other sources of information or comments on your response:

5. How confident are you in taking actions to reduce smoke impacts on your health?

- ☐ I am confident *I know how to protect myself* from harmful health impacts of smoke
- ☐ *I have some knowledge about actions to take*, but I am not confident I can reduce smoke impacts on my health
- ☐ *I have little knowledge about what actions to take* and I am not confident I can reduce smoke impacts on my health
- ☐ *I have no knowledge about what actions* to take and I am not confident I can reduce smoke impacts on my health
- ☐ I don't feel my health is impacted by smoke so I don't feel I need more information

6. Have you taken any of these actions to protect your health and lessen the smoke you breathe from prescribed fire smoke, wildfire smoke, or both? Check all that apply. **If you took action but do not know the source of smoke, check 'unsure about source of smoke.'**

**Action that I took:**	For prescribed fire smoke	For wildfire smoke	Unsure about source of smoke
used a face mask (e.g. cloth, **not** N95)	☐	☐	☐
used a respirator mask (N95 and similar)	☐	☐	☐
used a **purchased** air cleaner/purifier in my home	☐	☐	☐
used a **homemade** air cleaner/purifier in my home	☐	☐	☐
avoided daily activities, such as going out to the store	☐	☐	☐
avoided normal outdoor recreation	☐	☐	☐
stayed indoors	☐	☐	☐
left the area impacted by smoke	☐	☐	☐
ran my air conditioning	☐	☐	☐
used additional medication (example, for asthma)	☐	☐	☐

Please explain the main reasons why you did or did not take these actions, or other explanations:

7. Please tell us what types of information you would like about ***prescribed fire***.
Check all that apply.

- ☐ Advance warning (such as 1 week) about when prescribed fire may be near me
- ☐ Advance warning (such as 1-2 days) about when prescribed fire may be near me
- ☐ Advance warning about time of day when prescribed fire smoke may be in my area
- ☐ How to find air quality information and forecast on my smartphone or computer (Air Quality Index, AQI)
- ☐ Detailed information about the prescribed fire – number of acres, reasons for prescribed fire, who is conducting it, their contact information
- ☐ Health information, such as health risks for populations with various medical conditions
- ☐ Where to get respirator masks (N95 or similar) during prescribed fire events
- ☐ How and when to use a respirator mask (N95 or similar) during prescribed fire events
- ☐ How to set up a clean air room inside my home with a portable air filter (where to get one, how to choose the right one for me)
- ☐ How to make a home air filter using a box fan and adding filters (do-it-yourself, DIY)
- ☐ How to access a clean air shelter in my community
- ☐ Information about temporary relocation from smoky areas during prescribed fire events
- ☐ Other protective actions, such as how to improve air filtration (HVAC) in my home
- ☐ Information about prescribed fire benefits and safety
- ☐ No information needed

Other types of information or comments on your response:

8. If a fire starts unintentionally, but in moderate weather conditions and in an area where it would be helpful (such as where it could reduce future wildfire risk or improve forest health), *would you support having fire agencies manage the fire in a controlled manner* to achieve a beneficial purpose, rather than immediately working to extinguish it?

☐ Yes
☐ Maybe, but I'd like to have more information
☐ No
☐ I don't know

9. Do you support California's policy change to *increase the use of beneficial prescribed fire* as a means of reducing the risk of major wildfires and improving ecosystems?

☐ Yes, I support this policy change.
☐ I could support this, but I want to know more or have other reservations.
☐ No, I don't support this.
☐ I don't know.

PART III – Health and background information

10. Has a physician or healthcare provider ever told you that you have any of the following?

☐ Asthma
☐ Chronic Obstructive Pulmonary Disease (COPD)
☐ Other respiratory disease
☐ Hypertension (high blood pressure) or other heart disease
☐ Type II diabetes, metabolic syndrome, or obesity
☐ Allergies or other conditions related to the upper respiratory tract, eyes, and ears
☐ Other chronic disease
☐ None
☐ Prefer not to say

11. Have you experienced any of the following health symptoms when the outside air is smoky? Please indicate whether the smoke was during a wildfire, prescribed fire, or mark ‘unsure about source of smoke’ if you do not know the source of smoke. **Check all that apply**.

Symptom	Prescribed fire smoke	Wildfire smoke	Unsure about source of smoke	I do not experience these symptoms when it is smoky outside
**Respiratory Symptoms**, *such as runny or stuffy nose, scratchy throat, irritated sinuses, coughing, trouble breathing normally, shortness of breath, wheezing, asthma attack, allergic symptoms, or similar*	☐	☐	☐	☐
**Eyes and Ears**, *such as stinging, itchy, or watery eyes, ear infections, allergic symptoms, or similar*	☐	☐	☐	☐
**Cardiovascular**, *such as fast or irregular heart rate, pain or tightness in the chest, high blood pressure, or similar*	☐	☐	☐	☐
**Other**, *such as tiredness, dizziness, viral infections, or other*	☐	☐	☐	☐

If you experienced any of the above symptoms from breathing smoke, did you see a doctor or get medical care for those symptoms? ☐ Yes ☐ No ☐ N/A, no symptoms

Please add any comments if you would like to clarify your response:

Please tell us about yourself.

12. Please select your age group:

☐ 18-29	☐ 50-64	☐ ≥ 80
☐ 30-49	☐ 65-79	☐ Prefer not to say

13. Please select your gender:
    - ☐ Female
    - ☐ Male
    - ☐ Other/Not listed/Prefer not to say

14. How many years have you lived at your current residence?
    - ☐ Less than a year
    - ☐ 1-4 years
    - ☐ 5-9 years
    - ☐ 10-19 years
    - ☐ 20+ years
    - ☐ Prefer not to answer

15. What else would you like us to know concerning your thoughts about prescribed fire or your particular health and safety situation?

Please select which gift card you prefer. Gift cards will be provided by mail within four weeks of receipt of your completed survey:

- ☐ $25 Pioneer Market
- ☐ $25 RiteAid
- ☐ Prefer not to receive a gift card

Thank you for participating in the survey!

This research study, Public Health Impact of Prescribed Fire ("PHIRE"), is being led by the California Department of Public Health (CDPH) and can be contacted at (510) 620-3620.

It is separate from Mariposa County Public Health and the SAFE Program. If you would like to get in touch with SAFE, please call Mariposa County at (209) 966-3689.
